# Promoting structural sustainable design through the influence of quality control assessments

**DOI:** 10.1038/s41598-026-42152-4

**Published:** 2026-03-06

**Authors:** Til Lux, Tânia Feiri, Jan Philip Schulze-Ardey, Josef Hegger, Martin Claßen, Marcus Ricker

**Affiliations:** 1 Chair of Structural Concrete, TU Dortmund University, August-Schmidt-Str. 8, 44227 Dortmund, Germany; 2 Institute of Structural Concrete, RWTH Aachen University, Mies-van-der-Rohe-Str. 1, 52074 Aachen, Germany

**Keywords:** Conformity control assessment, Stochastic models, Building material properties, Filtering effect, Structural reliability, Engineering, Materials science

## Abstract

In structural reliability assessments, the selection of suitable parameters for the definition of stochastic models of component properties—such as concrete compressive strength, steel yield strength or geometric dimensions—is a prime requirement. Typically, during the production of structural components, several conformity control criteria, which are part of quality control assessments, are adopted to evaluate whether their properties comply with specified requirements. Previous investigations have demonstrated that the consideration of conformity control assessments in reliability studies might have a positive influence on the structural reliability of a component, thereby, enabling more material and resource efficient designs than conventional designs that do not take conformity control into account. In this investigation, a methodology grounded on the principles of reliability theory and Bayesian statistics is offered to quantify the positive effects of conformity control assessments in structural reliability levels. The practical application of this methodology is further demonstrated through an example extracted from previous investigations concerning the reliability level of a short concrete column subjected to compression. The results suggest that existing safety margins can be activated to adjust partial safety factors on the resistance side and, thus, optimise design solutions. Finally, possible improvements for the overall methodology are identified, opening avenues for the design of more sustainable structures.

## Introduction

In structural engineering, material and geometric properties play a major role on the mechanical performance and durability of structural components and systems^1,2^. The control of such properties during the execution (or production) of key structural materials (e.g., concrete, reinforcing steel) is ensured through conformity control assessment as part of quality control procedures. The goal of conformity control assessment is to compel contractors or producers to deliver high quality components and, thus, avoid rejection of the produced structural components^3–6^. In principle, structural components that are subjected to a conformity control inspection have improved properties (e.g., concrete compressive strength, steel yield strength or geometric dimensions) compared to those that do not go through such an assessment^3–7^.

**Fig. 1:**
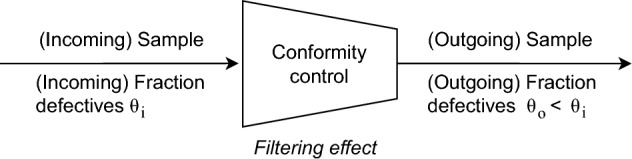
Illustration of the filtering effect from conformity control assessment (adapted from^3^).

The pioneering work of Rackwitz et al.^6–9^ conducted during the 1970s and 1980s laid important foundations to understand the impact of conformity control assessment on the reliability level of structural components. These Authors demonstrated that uncertainties in the actual distribution parameters (i.e., in terms of probability distributions) of concrete compressive strength can be meaningful and, thus, may lead to consequences on the reliability level of a component. Within this work, a general model was proposed to define the distribution of materials strength and consider the favourable effect of conformity control assessment on concrete compressive strength by means of Bayesian statistics^6^. Over the recent years, this knowledge was extended and consolidated in the work of Caspeele et al.^3–5^ through the quantification of a so-called filtering effect, whose concept is illustrated in Fig. 1. In a nutshell, the concept is the following: When components are subjected to a conformity control assessment and, then, inspected lots are accepted or rejected, the average quality of outgoing lots (i.e., after acceptance by conformity control) are higher than the average quality of incoming lots (i.e., presented for conformity assessment)^3–5^. This filtering effect influences the stochastic characterisation of concrete compressive strength.

To quantify the filtering effect, the incoming (i.e., original) statistical distribution of the (entire) population of the property—in this case, concrete compressive strength—can be updated into an outgoing (i.e., updated) statistical distribution of the accepted inspected lots^3–5^. In principle, due to this filtering effect from conformity control inspection, the scatter of the outgoing distribution is reduced and the expected value is ameliorated (i.e., the expected values have a tendency to increase). Then, when the outgoing statistical distribution of such parameters is considered in structural reliability assessments, the reliability level derived with the outgoing distribution might be higher than the reliability level derived with the incoming distribution (i.e., distribution without the contribution of quality control). The potential of the filtering effect identified by Caspeele et al.^3–5^ is, nowadays, particularly relevant to engineering design since it may enable the identification and further activation of hidden safety margins (or reserves). When safety margins exist, partial safety factors can be adjusted without compromising target reliability levels that are recommended in modern design codes as in EN 1990:2023^10^ (e.g., reliability index $\beta$ = 3.8, for a 50-year reference period and a reliability class RC 2 recommended for residential buildings and offices). The activation of those reserves may facilitate resource and material design optimisations^11–13^ and, thus, the reduction of greenhouse gas emissions of structural solutions—an ambitious goal of the building and construction sectors across the World^14^.

Albeit the groundbreaking nature of such preliminary work, in practice, not only concrete compressive strength is subjected to conformity control assessment. Instead, other material or geometric properties (e.g., as geometric dimensions, effective depth or, even, yield strength) that are also subjected to such control may influence the structural resistance level. The effect of conformity control assessment when it is concurrently performed on multiple properties was not considered in those preliminary studies. In this context, this investigation offers a novel methodology that enables the consideration and quantification of parallel conformity control assessments on distinct basic variables and open avenues to utilise such benefits to reduce partial safety factors without compromise predefined target reliability levels offered in modern structural codes, as in^10^. The underlying goal is that the activation of safety margins through the reduction of partial safety factors may promote material and resources efficiencies in early design stages. Furthermore, the stochastic models of concrete compressive strength utilised in those preliminary investigations mostly follow a Normal or a Lognormal distribution. Recent investigations conducted by Feiri et al.^15^ have demonstrated that a Log-Student-*t* distribution enables an accurate stochastic description of concrete strength. By utilising a Log-Student-*t* distribution, the distribution parameters can be Lognormal-Gamma-distributed, which is an advantage for the computation of the Bayes’ Theorem (part of Bayesian statistics) further detailed in Section “Brief overview on the fundamentals of Bayesian statistics”^3,6^. This enhanced stochastic model was also considered in the present investigation.

This manuscript is organised as follows: Sect. “Brief overview on the fundamentals of Bayesian statistics” provides an overview on the fundamentals of Bayesian statistics. Section “Methodology to consider the filtering effect of conformity control in reliability-based assessments” describes the methodology to consider the filtering effect of conformity control in reliability-based assessments. The methodology is applied to a numerical example described in Sect. “Numerical example”. Finally, Sect. “Conclusions” includes the main conclusions of this investigation and offers new paths for future research work.

## Brief overview on the fundamentals of Bayesian statistics

As briefly introduced in Sect. “Introduction”, Bayesian statistics form a suitable basis to update the distributional parameters of material or geometric properties, enabling the quantification of the filtering effect of conformity control, which can ultimately be considered in structural reliability assessments^3–9^. The underlying principle is that the parameters of a distribution for a basic variable *X* (e.g., concrete compressive strength, steel yield strength or geometric properties) are assumed as random variables. For the cases that these parameters are described through a two-parameter distribution (e.g., Normal or Lognormal distribution), both parameters of the distribution—i.e., the expected value $\mu$ and the standard deviation $\sigma$—correspond to Normal or Lognormal distributions. Since the parameters μ and σ are regarded as random variables, they can be described by a joint density function ${f}_{\mathrm{M},\Sigma }(\mu ,\sigma )$, with Μ referring to the mean and $\Sigma$ referring to the standard deviation of the distribution function. Both parameters Μ  and $\Sigma$ are associated to the conformity control criteria under consideration. In the following, all the formulae are valid for a normally-distributed (i.e., with two parameters) basic variable *X*.

The prior and posterior joint density functions refer to the functions before and after conformity control assessment, i.e., ${f'}_{\mathrm{M},\Sigma }(\mu ,\sigma )$ and ${f''}_{\mathrm{M},\Sigma }(\mu ,\sigma )$, respectively. The Bayes’ Theorem—one of the cornerstones of Bayesian statistics—requires a Likelihood function ${L}(\mu ,\sigma |{I})$ to update a prior function with additional information *I* into a posterior function. Additional information can be directly considered (e.g., experimental test values on similar structures or components) or indirectly considered (e.g., findings reported in literature and/or expert input or engineering judgement). The posterior probability density function ${f''}_{\mathrm{M},\Sigma }(\mu ,\sigma )$ expressed through Eqs. (1)—known as the Bayes’ Theorem—is strongly dependent on ${f'}_{\mathrm{M},\Sigma }(\mu ,\sigma )$ and ${L}(\mu ,\sigma |{I})$^16^:

1 $$\begin{aligned} f''_{\mathrm{M},\Sigma }(\mu ,\sigma ) = \frac{f'_{\mathrm{M},\Sigma }({\mu ,\sigma }) \cdot L(\mu ,\sigma |I) }{ \iint f'_{\mathrm{M},\Sigma }({\mu ,\sigma }) \cdot L(\mu ,\sigma |I) \, d\mu \, d\sigma } \end{aligned}$$

It should be highlighted that the choice of prior distribution in combination with the weight of the Likelihood function (i.e., here defined through experimental data) affects the posterior distribution. Considerations on the possible trends followed by these distributions are described in detail in^16,17^.

## Methodology to consider the filtering effect of conformity control in reliability-based assessments

**Fig. 2:**
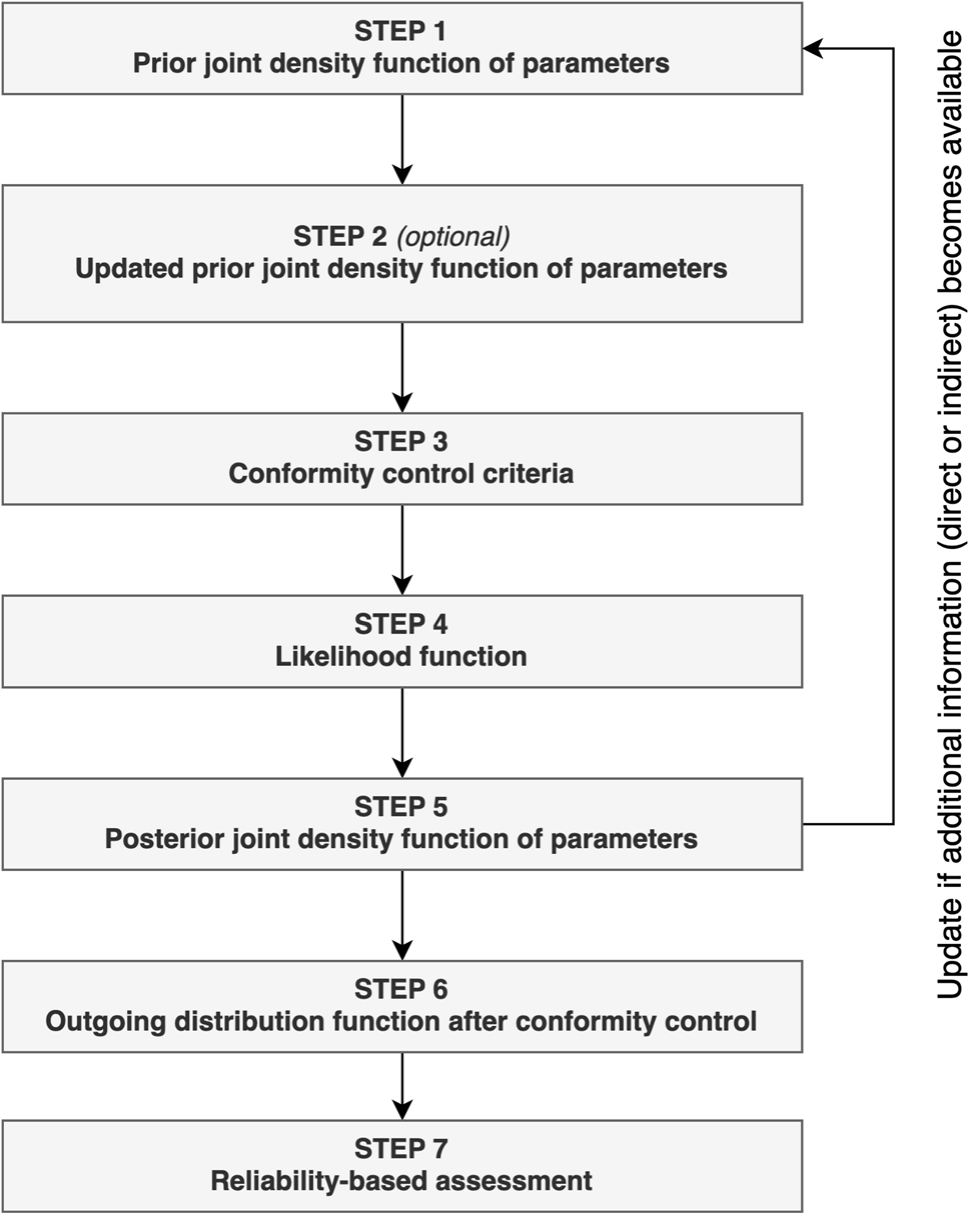
Methodology to update the distributional parameters of properties and integrate them into reliability-based assessments (adapted from^17^).

Based on a preliminary work presented in^17^, a methodology is proposed to consider the filtering effect of conformity control in reliability-based assessments (Fig. 2).

*Step 1. Prior joint density function* ${f'}_{\mathrm{M},\Sigma }(\mu ,\sigma )$ *of parameters*

In this step, suitable prior information should be selected and characterised for the stochastic characterisation of the property under analysis. Examples of such properties are concrete compressive strength and steel yield strength as well as geometric properties as dimensions and effective depth. Prior information can be retrieved from reference literature such as JCSS Probabilistic Model Code^18^. For example, Table 1 lists the prior hyperparameters *m’*, *n’*, *s’* and $\nu$’ available in JCSS Probabilistic Model Code for the stochastic characterisation of ready-mixed concrete according to distinct concrete strength classes. Prior information on concrete compressive strength can be found in other relevant literature or can be entirely tailor made based on the type of information available. For example, in^15^ a set of revised prior hyperparameters were proposed, which were derived on the basis of a large dataset of experimental strength results available in Germany. Also, a set of generalised prior parameters based on combined vague-informative prior information is offered in^19^ to be utilised when the number of samples is limited.

Unlike the prior information available for concrete compressive strength, prior information for basic variables is not always available. Typically, this is the case of steel yield strength or geometric properties. In the absence of documented prior information, synthetic data can be generated providing that it follows the stochastic nature of the property being investigated. To this, also the JCSS Probabilistic Model Code offers a wide range of stochastic models for the most relevant basic variables. Also the recently introduced guideline “Procedure for the derivation of safety factors in concrete structures using probabilistic methods” developed by the German Committee for Structural Concrete (DAfStb) includes a set of stochastic models for relevant basic variables^20^. Additionally, a wider range of literature including stochastic models for relevant basic variables is available as, e.g.,^1,21–24^. Once data is generated, the so-called Maximum Likelihood Estimators (MLE) can be utilised to derive new prior parameters for the function ${f'}_{\mathrm{M},\Sigma }(\mu ,\sigma )$. The derivation of such estimators is further detailed, for example, in^6,25^.

**Table 1:** Prior hyperparameters for concrete strength distribution for ready-mixed concrete according to JCSS Probabilistic Model Code^18^.

Concrete grade	Prior hyperparameters$^{(*)}$			
	*m’*	*n’*	*s’*	$\nu '$
C15	3.40	3.0	0.14	10
C25	3.65	3.0	0.12	10
C35	3.85	3.0	0.09	10
C45	3.98	3.0	0.07	10
C55	–	–	–	–

*m’*: mean value of an equivalent sample of size *n*’.

*s’*: standard deviation of an equivalent sample of size $\nu$’+1.

$\nu$’: degrees of freedom.

$^{(*)}$ Hyperparameters for cylindric samples (reference dimensions: 300 mm height and 150 mm diameter)

*Step 2. Updated prior joint density function* ${f'}_{\mathrm{M},\Sigma }(\mu ,\sigma )$ *of parameters (optional)*

**Fig. 3:**
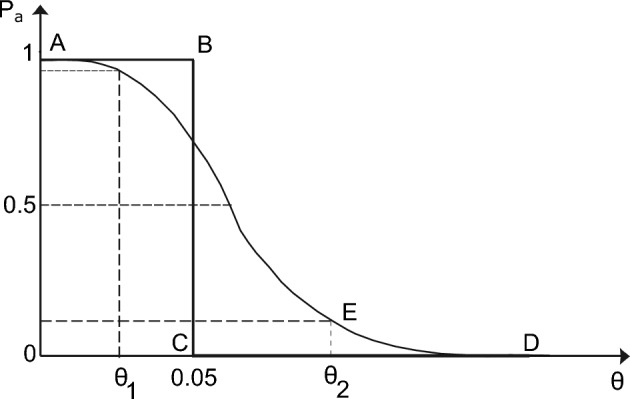
Generic form of an OC curve^3,5,15,26,27^.

If experimental data is available (e.g., measured strength values or measured geometric dimensions), the prior joint density function ${f'}_{\mathrm{M},\Sigma }(\mu ,\sigma )$ can be directly updated. More, if such prior information is available in the direct form of data pairs of the sample parameters ${m}_{i}$ and ${s}_{i}$ from *k* samples, MLE^6,25^ can be utilised to update prior parameters for ${f'}_{\mathrm{M},\Sigma }(\mu ,\sigma )$. As mentioned in Step 2, the derivation of such estimators is further detailed in^6,25^.

*Step 3. Conformity control criteria*

In this step, specific conformity criteria shall be selected for the control of material and geometric properties. These criteria can be retrieved from codes of practice, standards or even country-specific regulation (e.g., for concrete compressive strength in DIN EN 206:2021^28^ and in DIN 1045-2^29^, for reinforcing steel in DIN EN 10080:2005^30^ and DIN 488-6:2024^31^ and for geometric properties EN 13670:2011^32^, and DIN 18202:2019^33^, among others). Notwithstanding, bespoken conformity control criteria can be also established based on specific conformity control requirements.

*Step 4. Likelihood function* ${L}(\mu ,\sigma |{I})$

The goal of this step is to determine the Likelihood function ${L}(\mu ,\sigma |{I})$ needed for the Bayes’ Theorem expressed through Eq. (1). When conformity control criteria are utilised as described in Step 3, the quantification of the filtering effect is rather straightforward through Operating Characteristics (i.e., OC functions or curves) describing the discriminating capacity of the adopted criteria. An OC function ${P}_{a}(\theta )$ illustrates the probability ${P}_{a}$ that an inspection lot characterised by a certain fraction defective $\theta$ is accepted^3,5,15,26,27^. The term $\theta$ refers to the fraction of the population below a specified Acceptance Quality Level (also known as AQL) (e.g., the 5%-quantile of the theoretical distribution of concrete compressive strength)^34^. An ideal OC function of a “perfect” conformity control (i.e. no wrong decisions occur), which allows for a fraction defectives of 5% (e.g., for material strengths), is represented by the lines ABCD (Fig. 3). This can only be achieved if each individual production unit is subjected to conformity control or if the hypothetical sample is infinitely large for continuous processes in which the product cannot be discretely separated. For basic conformity criteria, analytical expressions are available to compute the function ${P}_{a}(\theta )$^6,15^. When combined or complex conformity criteria are needed, Monte Carlo simulations can support the computation of ${P}_{a}(\theta )$^3,5^.

For those properties that are characterised by an attribute, such as geometric dimensions or effective depth, the probability of acceptance ${P}_{a}(\theta )$ follows a distribution function (Bernoulli type), which is suitable for independent samples^34,35^. In this case, the probability that in a *n*-sample taken from a lot will have at most *k* defective items can be estimated as:

2 $$\begin{aligned} P_{a}(\theta ) = {n\atopwithdelims ()k} \theta ^{k} \cdot (1 - \theta )^{n - k} \end{aligned}$$

with ${k} = 0,1\ldots$, *n*. The probability of acceptance *P*_*a*_(θ) of a defective lot *θ* in the *k*∥*n* plan is called the characteristic of a single plan and the graph of dependency on *θ* is the OC function (or curve). Further explanation regarding the utilisation of OC functions for the conformity control of geometric properties is available in^34,35^.

*Step 5. Posterior joint density function* ${f''}_{\mathrm{M},\Sigma }(\mu ,\sigma )$ *of parameters*

Here, the posterior joint density function ${f''}_{\mathrm{M},\Sigma }(\mu ,\sigma )$ shall be determined on the basis of Equation 1 through the combination of the prior function ${f'}_{\mathrm{M},\Sigma }(\mu ,\sigma )$ and the Likelihood function ${L}(\mu ,\sigma |{x})$. Frequently, this step requires the utilisation of numerical simulation methods^3,6^.

*Step 6. Outgoing distribution function after conformity control*

Once the effect of conformity control criteria is quantified, the predictive distribution of the material or geometric property (i.e., basic variable *X*) can be estimated. By integrating over the parameters $\mu$ and $\sigma$, the desired distribution of *X*—i.e., outgoing distribution function for the material or geometric property—can be determined as the marginal function ${f}_{X}({x})$ of the posterior density function ${f''}_{\mathrm{M},\Sigma }(\mu ,\sigma )$:

3 $$\begin{aligned} \textit{f}_{X}(x) = \int _{0}^{\infty} \int _{-\infty}^{\infty} {f}_{X}(x|{\mu ,\sigma }) \cdot {f''}_{\mathrm{M},\Sigma }({\mu ,\sigma }) \, d\mu \, d\sigma \end{aligned}$$

When a basic variable *X* is Normally or Lognormally distributed, the distribution for $\mu$ and $\sigma$ can be described by a Normal-gamma or Lognormal-gamma distribution and the marginal density is a Student’s-*t* or Log-Student’s-*t* distribution^15,17^. This function shall be further considered in reliability-based assessments^6^.

*Step 7. Reliability-based assessment*

This step aims to assess the influence of conformity control assessment on the level of structural reliability. To this, reliability-based techniques, such as First- or Second Order Reliability Methods (i.e., FORM or SORM) and/or Monte Carlo simulations—Crude or with variance reduction techniques as Importance Sampling or Subset Sampling—can be utilised^1,36^. Robust statistical software tools are available such as those described in^36–40^.

## Numerical example

### General considerations

In this section, an axially loaded reinforced concrete column with square section under compression load was extracted from^3,4^ and analysed with the aim to demonstrate how the described methodology can be applied to the resolution of common structural engineering problems. For this purpose, the limit state function ${g}({\overrightarrow{x}})$ of a column failure under compression is expressed as^3,4^:

4 $$\begin{aligned} \textit{g}({\overrightarrow{x}}) = \theta _{R} \cdot \left[ (1 - \rho _{l}) \cdot \textit{b} \cdot \textit{h} \cdot \alpha _{cc} \cdot \textit{f}_{c} + \rho _{l} \cdot \textit{b} \cdot \textit{h} \cdot \textit{f}_{y} \right] \, - \, \theta _{E} \cdot (\textit{G} + \textit{Q}_{50}) = 0 \end{aligned}$$

with the basic variables and respective stochastic models being described in Table 2.

To design a reinforced concrete member exposed to permanent and imposed load effects, EN 1990:2011^41^ provides the fundamental load combination for an accompanying action *Q* (e.g., Category A imposed load in building construction)^3,4^:

5 $$\begin{aligned} \begin{aligned} \textit{R}_{d} = \text {max} \{ \gamma _{G} \cdot \textit{G}_{k} + \psi _{0,Q} \cdot \gamma _{Q} \cdot \textit{Q}_{k}; \, \xi \cdot \gamma _{G} \cdot \textit{G}_{k} + \gamma _{Q} \cdot \textit{Q}_{k} \} =\\ \text {max} \{ 1.35 \cdot \textit{G}_{k} + 0.7 \cdot 1.5 \cdot \textit{Q}_{k}; \, 0.85 \cdot 1.35 \cdot \textit{G}_{k} + 1.5 \cdot \textit{Q}_{k} \} \end{aligned} \end{aligned}$$

For an economic design where the design value of the load effect corresponds to the design value of the resistance, ${G}_{k}$ and ${Q}_{k}$ can be calculated for a given resistance ${N}_{Rd}$ and a given load ratio χ:

6 $$\begin{aligned} \begin{aligned} \chi = \frac{\textit{Q}_{k}}{\textit{G}_{k} +\textit{Q}_{k}} \\ \end{aligned} \end{aligned}$$

The influence of conformity control on ${f}_{c}$ and on *b* and *h* were investigated. The computational analysis was conducted in TesiproV^37^, an open-source software library developed in *R*^42^. The reliability-based assessment utilised a FORM method (Hasofer-Lind algorithm^43^).

**Table 2:** Probabilistic models for the basic variables based on^3,4,18^.

*X*	Basic variable	Dist. type	Nominal value ${X}_{k}$	Expected value $\mu _{x}$	Stand. deviation $\sigma _{x}$
${f}_{c}$	Concrete compressive strength (MPa)$^{(a)}$	LST	${f}_{ck}$	Equation I	Equation II
${f}_{y}$	Yield strength of reinforcing steel (MPa)	N	${f}_{yk}$	500 + 2 $\cdot$ $\sigma_{x}$	30
*b*	Width (mm)$^{(b)}$	ST	${b}_{nom}$	${b}_{nom}$ + 0.003 $\cdot$ ${b}_{nom}$	4 + 0.006 $\cdot$ ${b}_{nom}$
*h*	Height (mm)$^{(b)}$	ST	${h}_{nom}$	${h}_{nom}$ + 0.003 $\cdot$ ${h}_{nom}$	4 + 0.006 $\cdot$ ${h}_{nom}$
$\rho _{l}$	Reinforcement ratio (−)	N	$\rho _{l,nom}$	$\rho _{l,nom}$	0.02 $\cdot$ $\rho _{l,nom}$
*G*	Permanent load (MN)	N	${G}_{k}(\chi ,{N}_{Rd})$	${G}_{k}$	0.1 $\cdot$ ${G}_{k}$
${Q}_{50}$	Imposed load (MN)	GU	${Q}_{50,k}(\chi ,{N}_{Rd})$	0.6 $\cdot$ ${Q}_{k}$	0.35 $\cdot$ 0.6 $\cdot$ ${Q}_{k}$
$\theta _{R}$	Resistance uncertainty (−)	LN	–	1.0	0.1
$\theta _{E}$	Load effect uncertainties (−)	LN	–	1.0	0.1

LST: Logstudent’s *t*-distribution; N: Normal distribution; LN: Lognormal distribution; ST: Student’s *t*-distribution; GU: Gumbel distribution

^(a)^The prior hyperparameters of the distribution (Normal-Gamma) describing the distribution of the paramaters of concrete compressive strengths (${m}^{\prime},{n}^{\prime},{s}^{\prime}$ and $\nu ^{\prime}$) were interpolated from the prior information available in JCSS Probabilistic Model Code^18^. The expected value (Equation I) and standard deviation (Equation II) can be calculated depending on these parameters: Equation I: $\mu _{Y}$ = e$^{m^{\prime}+0.5\cdot s^{\prime2}}$; Equation II: $\sigma _{Y}$ = e$^{m^{\prime}+0.5\cdot s^{\prime2}}$ $\cdot$ $\sqrt{e^{m^{\prime} + 0.5\cdot s^{\prime2}} - 1 }$

$^{(b)}$ The prior hyperparameters describing the parameters of the Student’s *t*-distribution of the external dimensions were calculated using MLE^6,25^ for a synthetic dataset with a sample size of *N* = 10 000 and a random sample size of *n* = 15. The synthetic samples were generated using random values from a normally-distributed population with the above-listed stochastic parameters.

### Analysis and results

*Step 1. Prior joint density function* ${f'}_{\mathrm{M},\Sigma }(\mu ,\sigma )$ *of parameters*

In Step 1, the prior joint density functions ${f'}_{\mathrm{M},\Sigma }(\mu ,\sigma )$ for the properties under analysis—i.e., concrete compressive strength and geometric dimensions—were defined. The information for the prior distribution of concrete compressive strength was retrieved from the JCSS Probabilistic Model Code^18^. For the geometric dimensions, synthetic data was generated fitting a probabilistic model given in JCSS Probabilistic Model Code since neither a prior distribution is offered in reference literature nor experimental data is available as it is detailed in Table 2.

*Step 2. Updated prior joint density function* ${f'}_{\mathrm{M},\Sigma }(\mu ,\sigma )$ *of parameters (optional)*

Step 2 was excluded from this numerical analysis since experimental data was not available.

*Step 3. Conformity control criteria*

In this step, conformity control criteria were selected for the properties under analysis. For the concrete compressive strength, the criteria were extracted from DIN EN 206:2021^28^ considering a continuous production regime with Criterion 1 being ${f}_{cm}$ $\ge$ ${f}_{ck}$ + 1.48 $\cdot$ $\sigma$ and Criterion 2 being ${f}_{ci}$ $\ge$ ${f}_{ck}$ – 4. Here, ${f}_{cm}$ refers to the mean value of concrete compressive strength samples of a production batch, ${f}_{ck}$ refers to the concrete compressive strength of the target strength class and ${f}_{ci}$ refers to the individual value of concrete compressive strength sample of a production batch.

**Fig. 4:**
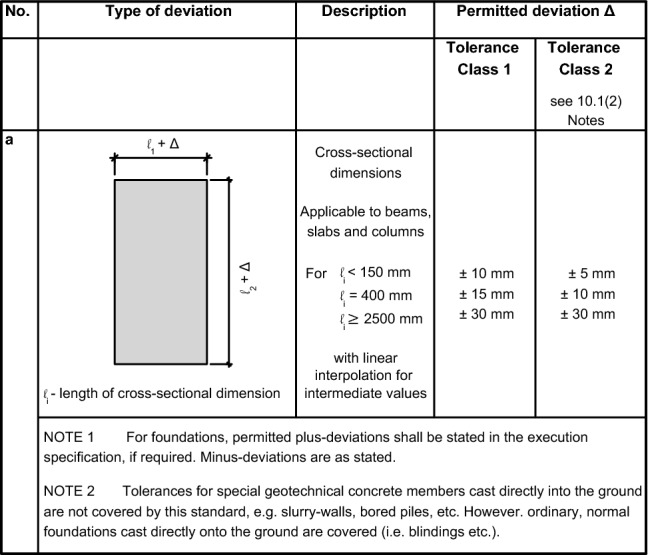
Extract of conformity control criteria for cross-sectional properties based on DIN EN 13670:2011^32^.

For the geometric dimensions, the provisions of DIN EN 13670:2011^32^ were considered concerning to permitted deviation by means of Tolerance Classes 1 and 2 (Fig. 4).

**Fig. 5:**
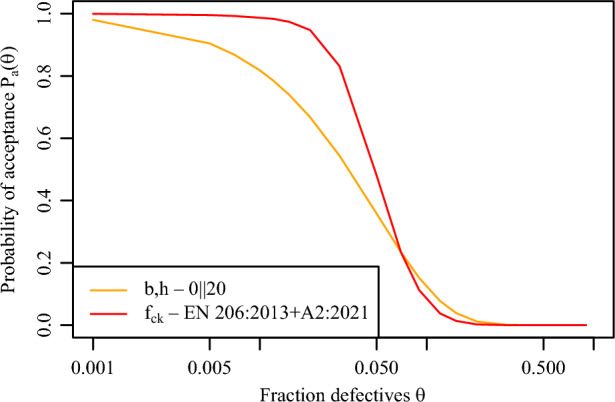
OC curves for the concrete compressive strength and for the geometric properties (sampling plan 0 || 20).

*Step 4. Likelihood function* ${L}(\mu ,\sigma |{I})$

In this step, the Likelihood functions ${L}(\mu ,\sigma |{I})$ needed in Eq. (1) were estimated through OC functions. Figure 5 illustrates the OC functions for both properties under analysis. For the concrete compressive strength, since both multiple conformity control criteria needed to be evaluated (Step 4), the OC function required as the Likelihood function ${L}(\mu ,\sigma |{I})$ could not be analytically determined. Therefore, the probability of acceptance ${P}_{a}$($\theta$) for discrete points $\theta$ was approximated in a Monte Carlo simulation. This involved generating *N* = 100 000 fictitious samples (each with *n* = 15 test results), originated from a given distribution and parameters of the distribution utilised with the respective proportion of defective units $\theta$. Then, it was assessed whether both conformity criteria were met. The ratio of the number of accepted samples to the total number of samples provided the approximate ${P}_{a}$($\theta$). Then, the points between the discrete $\theta$ values were interpolated. For the geometric properties, a sampling plan was idealised consisting of 20 inspected items in a batch and zero defective items (sampling plan 0 || 20) (Fig. 5). The OC function was determined through Eq. (2).

*Step 5. Posterior joint density function* ${f''}_{\mathrm{M},\Sigma }(\mu ,\sigma )$ *of parameters*

**Fig. 6:**
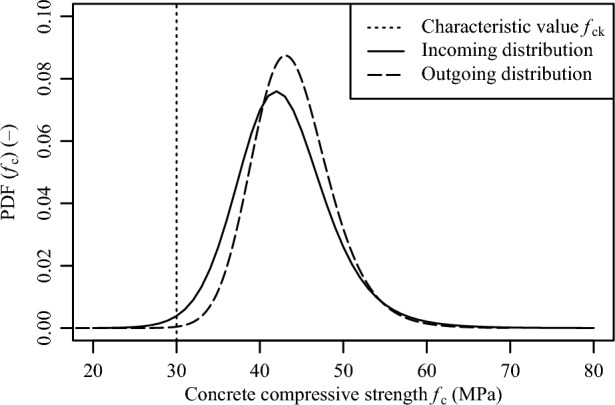
Incoming and outgoing distribution for the concrete compressive strength ${f}_{ck}$.

**Fig. 7:**
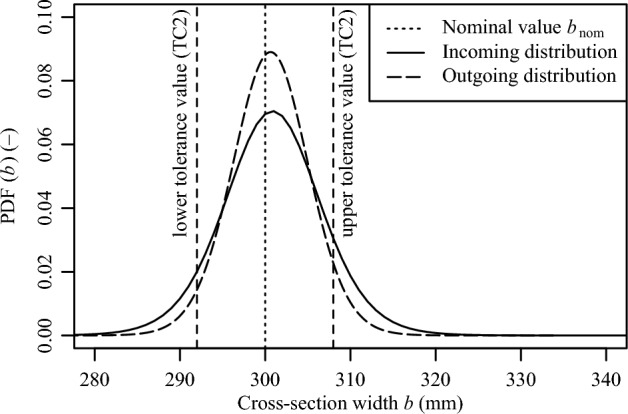
Incoming and outgoing distribution for the column width *b*.

In Step 5, the posterior joint density function ${f''}_{\mathrm{M},\Sigma }(\mu ,\sigma )$ was computationally determined according to Eq. (1).

*Step 6. Outgoing distribution function after conformity control*

In Step 6, the marginal function of the updated joint probability distributions was determined with Eq. (3). Figure 6 illustrates the distributions without and with conformity control (i.e., incoming and outgoing distributions, respectively) for an entire range of concrete compressive strengths ${f}_{ck}$ on the basis of DIN EN 206:2021^28^. Here, a continuous production was considered. For reference, a sample size of *n* = 15 values and concrete compressive strength ${f}_{ck}$ value of 30 N/mm$^{2}$ were assumed. Figure 6 demonstrates that due to conformity control, the standard deviation decreased from 5.81 N/mm$^{2}$ to 4.89 N/mm$^{2}$. The expected value followed an inverse trend by increasing from 42.8 N/mm$^{2}$ to 44.0 N/mm$^{2}$.

By using the OC functions for the Likelihood function in Eq. (1), the influence of conformity control assessment on the geometric properties can be quantified. Figure 7 illustrates the prior distribution (i.e., without conformity control assessment) and the posterior distribution (i.e., with conformity control assessment) for the cross-section width *b*. The positive effect of conformity control assessment led to a decrease of the standard deviation from 6.08 N/mm$^{2}$ to 4.69 N/mm$^{2}$. In this case, the expected values remain unchanged.

*Step 7. Reliability-based assessment*

**Fig. 8:**
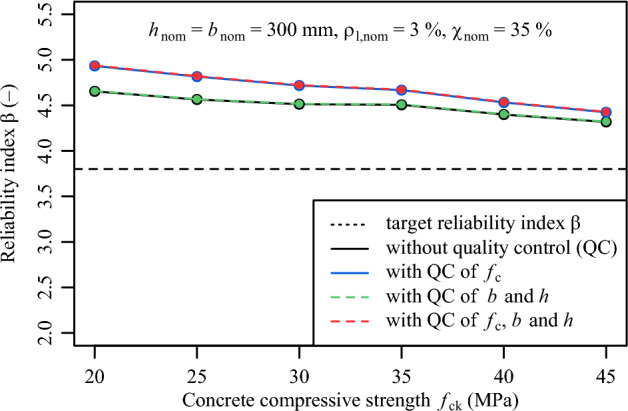
Variation of reliability index $\beta$ on concrete compressive strength ${f}_{ck}$ with and without the filtering effect of conformity criteria.

The reliability-based assessment conducted in Step 7 confirms the positive influence of conformity control assessment on the structural reliability level of the column being investigated for the entire range of concrete strength classes. To this, it was assumed that ${h}_{nom}$ and ${b}_{nom}$ is equal to 300 mm, $\rho _{l,nom}$ is equal to 3% and the load ratio ${\chi}_{nom}$ is equal to 35%. Figure 8 demonstrates that by utilising the outgoing distribution (i.e., after conformity control assessment), the reliability index $\beta$ results higher than the $\beta$ value determined with the incoming distribution (i.e., before conformity control assessment). Depending on the parameter combination, the reliability index $\beta$ increases from 3% up to 10% due to conformity testing in accordance with DIN EN 206:2021^28^.

**Fig. 9:**
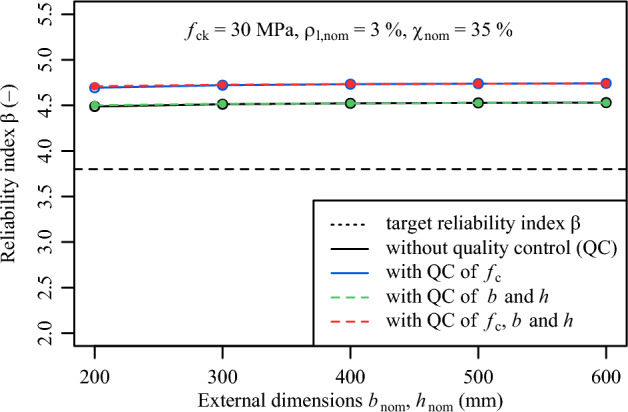
Variation of reliability index $\beta$ on geometric variables *b* and *h* with and without the filtering effect of conformity criteria.

A further investigation was conducted on the variation of the external dimensions when they are subjected to conformity control assessment. To this, it was assumed that ${f}_{ck,nom}$ is equal to 30 MPa, $\rho _{l,nom}$ is equal to 3% and the load ratio ${\chi}_{nom}$ is equal to 35%. From this investigation, the results suggest that conformity control on the external dimensions has a marginal increase on the $\beta$ value, which can be attributed to the small scatter of the external dimensions compared to the scatter from concrete compressive strength (Fig. 9). Since the scatter of the external dimensions has a minor influence on the overall scatter of the load-bearing capacity, a smaller scatter of the external dimensions has a minimal influence on the calculation of the failure area from which the probability of failures and reliability indices $\beta$ are determined.

**Fig. 10:**
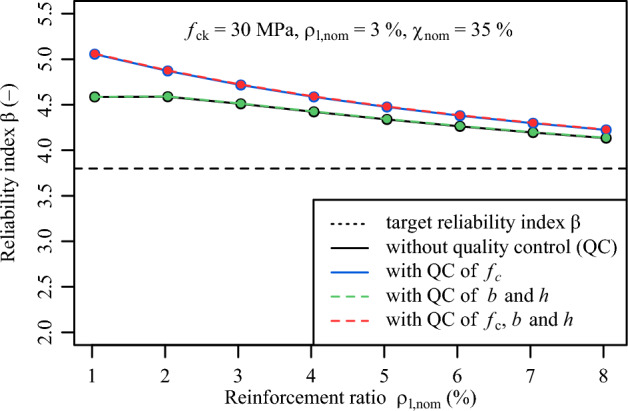
Variation of reliability index $\beta$ on reinforcement ratio $\rho _{l}$ with and without the filtering effect of conformity criteria.

Figure 10 illustrates the variation of the reliability indices $\beta$ for the reinforcement ratio $\rho _{l,nom}$ varying between 1% and 8%. From this illustration, it is visible that conformity control assessment has a positive influence on the entire range of reinforcement ratios $\rho _{l,nom}$. Yet, the influence is more prominent for small reinforcement ratios than for large reinforcement ratios. For example, for a $\rho _{l,nom}$ of 1% the $\beta$ value increases from 4.5 to 5.1, while for a $\rho _{l,nom}$ of 8%, the increase of the $\beta$ value is marginal from 4.2 to 4.3.

**Fig. 11:**
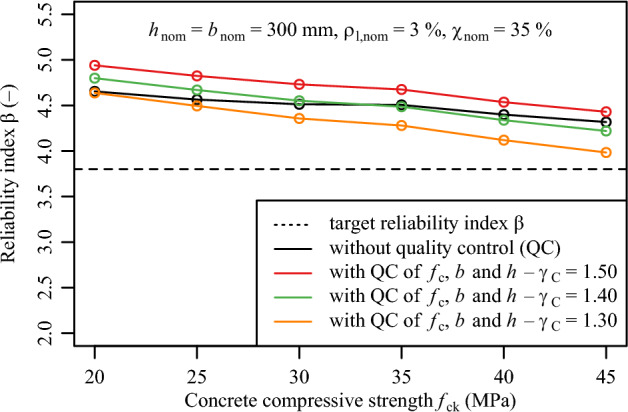
Variation of reliability index with the filtering effect of conformity criteria with respect to concrete compressive strength ${f}_{ck}$ and variation of partial safety factors for concrete $\gamma _{C}.$.

### Potential to reduce partial safety factors

The results discussed in Section 4.2 indicate that the effect of conformity control on concrete compressive strength leads to higher reliability indices $\beta$ than the target $\beta$ value of 3.8, the reference value recommended in EN 1990:2023^10^. These results suggest that safety margins exist and can be activated through the reduction of some partial safety factors.

Figure 11 illustrates the variation of $\gamma _{C}$ from the default value of 1.5. Additionally, it demonstrates that reducing the partial safety factor $\gamma _{C}$ to 1.40 for all the considered parameter combination leads to, at least, the same the reliability index $\beta$ attained without conformity control assessment. Further reduction of the partial safety factor $\gamma _{C}$ to 1.30 appears to be feasible if the conformity control criteria follow the provisions of DIN EN 206:2021^28^. Since the influence of the conformity control assessment on *b* and *h* on the outgoing reliability index $\beta$ appears to be negligible, conformity control on the variables *b* and *h* is, in this case, not necessary for a reduction of $\gamma _{C}$.

### Discussion on further opportunities to leverage structural reliability margins through the methodology

The findings of this investigation indicate that the influence of conformity control assessment on the reliability level of structural components varies according to the property being investigated. Additionally, it varies according to the selected conformity control criteria. This suggests that the positive effect of conformity control assessment may be even more pronounced if it is derived from stringent conformity control criteria. This influence might also vary according to the structural member and failure mode being analysed and, therefore, further investigations shall be conducted in this direction.

Another aspect to consider concerns the influence of autocorrelation. Previous studies suggest that autocorrelation is present when a large set of results of consecutive tests are conducted due to the manufacturing process since basic factors that contribute to the scattering of the material or geometric properties remain constant over a certain period of time. For example, autocorrelation of concrete compressive strength is due fundamental factors contributing to the variation of concrete compressive strength such as cement strength, moisture content, and aggregate particle size distribution. For other basic variables (e.g., geometric parameters), statistical methods (e.g., Durbin-Watson test^44,45^) must be utilised to evaluate whether autocorrelation effects need to be taken into account. The autocorrelation effect can be quantified, for example, by using a second order autoregressive time series model^3,46^.

From a practical perspective, the most prominent challenge affecting the efficiency of the described methodology concerns the lack of experimental data available to update the prior joint density function of the distribution parameters ${f'}_{\mathrm{M},\Sigma }(\mu ,\sigma )$. In the case of concrete compressive strength, the available prior information is based on strength data collected during the 1960s^6,47^. Yet, since the concrete technology has significantly progressed over the last few decades^48,49^, it can be assumed that the variation of modern concretes might be considerably lower than the one currently considered in reference documents. Thus, it is presumed that the estimated structural reliability might be somewhat conservative, i.e., the probabilities of failure and reliability indices may be overestimated. With more recent data, the calculations can be carried out with enhanced accuracy, thereby, increasing the potential to reduce partial safety factors. Similar limitations affect geometric data since open databases of geometric measurements are somewhat limited and, thus, prior information rely exclusively on artificially-generated data.

## Conclusions

The present investigation led to the following conclusions:

- A methodology based on a Bayesian statistics and reliability-based principles is offered enabling the quantification of the filtering effect of conformity control assessments, which can be further considered in reliability-based analyses of structural components.
- The practical application of the methodology was demonstrated through a numerical example. Depending on the assumptions adopted in this example, the results indicated the following:
  - The filtering effect of conformity control assessment on concrete compressive strength may increase the reliability level of the component up to 10%.
  - Even though conformity control on the geometric properties led to negligible effects in the outgoing reliability level, the influence of conformity control on the geometric accuracy (e.g., geometric dimensions, location of reinforcement) may, nevertheless, be more expressive in different structural problems.
  - This increase indicates that the partial safety factor for concrete $\gamma _{C}$ may be reduced from 1.50 to 1.30, thereby, suggesting that resources and material efficiencies might be feasible already in early design stages.
- The results suggest that the Bayes’ Theorem included in the stochastic approach remains a suitable basis to update prior distributions provided that additional information from conformity control assessment is available.

Further investigations should be conducted to overcome the limitations of the proposed methodology. To this, the influence of tailor made—and, if possible, stringent—conformity control criteria applied to multiple material and geometric properties shall be investigated. Also, mathematical optimisations of the assessment approach, such as the consideration of the autocorrelation effect, are idealised. Due to major challenges concerning in-situ data (e.g., geometric properties), the potential to integrate conformity control strategies, reliability-based assessments and modern digital tools shall continue to be studied. For the methodology, further investigations can be conducted on the sensitivity of the results to the choice of prior distributions. Outside the scope of the proposed methodology, the influence of conformity control criteria on structural components made of modern concretes (i.e., low carbon concrete) shall be addressed. The extent to which resources and material efficiencies can be attained shall be thoroughly evaluated alongside the quantification of greenhouse gas emissions. Finally, integrating time-dependent effects in the proposed methodology, thereby, enabling a lifecycle reliability of structural systems through frameworks as^50,51^ should be considered in future research activities.

## Data Availability

The authors declare that the data supporting the findings of this study are available within the paper.
